# Fall risk in relation to strength training, walking, and sitting among women aged 75–105 years: a cross-sectional study within the Women’s Health Initiative Strong and Healthy (WHISH) Intervention Cohort

**DOI:** 10.21203/rs.3.rs-7246621/v1

**Published:** 2025-08-13

**Authors:** Corey Rovzar, Saachi Jhandi, Sally Mackey, Deepika Laddu, Sa Shen, Andrea LaCroix, Marcia L. Stefanick

**Affiliations:** Stanford University School of Medicine; University of Utah School of Medicine; Stanford University School of Medicine; Northwestern University; Stanford University; University of California, San Diego; Stanford University School of Medicine

**Keywords:** Balance, balance confidence, PA, physical function, fall risk, falls

## Abstract

**Background:**

Falls are the leading cause of injury and injury death among older women in the United States. The Women’s Health Initiative Strong and Healthy (*WHISH*) trial intervention cohort provided the opportunity to assess how fall risk classification (FR-ABC), as measured by balance confidence, relates to strength training, walking, sitting, and physical function (PF) in women aged 75 and over, a growing population at particularly high fall risk.

**Methods:**

We analyzed data from 8,915 women aged 75–105 years (mean age 84.9 ± 5.1 years; 49.6% ≥85 years) randomly assigned to a PA intervention in the *WHISH* pragmatic trial. Participants self-reported their weekly participation in strength training, walking, daily sitting time, and current PF. Balance confidence was measured using the Activities-specific Balance Confidence (ABC) scale, with an ABC score of < 67% used to classify FR-ABC. We assessed FR-ABC prevalence in the total cohort and for the women aged 75–84 and 85 years and older and cross-sectional associations with strength training, walking, sitting, and PF categories using analysis of variance (ANOVA) and chi-squared tests.

**Results:**

Mean age was 84.9 years, with 49.6% of participants aged ≥ 85 years. FR-ABC classification was 35.1% overall, 22.7% for women aged 75–84 and 47.4% for women aged ≥ 85 years. Overall, strength training ≥ 2 hours per week was associated with a 34% FR-ABC risk reduction (RR = 0.66, 95% CI: 0.61–0.71); walking ≥ 4.25 hours/week with a 70% FR-ABC risk reduction (RR = 0.30, 95% CI: 0.27–0.33); while, sitting ≥ 8 hours/day was associated with a 41% increased FR-ABC risk (RR = 1.41, 95% CI: 1.33–1.49). High PF (score ≥ 90) was associated with a 91% FR-ABC risk reduction (p < 0.001).

**Conclusion:**

More strength training and walking, lower sitting time, and higher PF were strongly associated with reduced fall risk classification among women aged 75 years and older. Although these associations were more pronounced among women aged 75–84 year, it was also true for women aged 85 and over. Fall risk prevention should emphasize each of these PA factors.

## Introduction

Falls among older adults occur in 25% of those ≥ 65 and 50% of those ≥ 80 years old, resulting in annual costs of $80 billion in the US [1–4]. Older women are more likely to fall than older men and are also more prone to hip fractures with a fall, due to higher rates of osteoporosis [2]. Thus, there is a critical need for interventions that specifically reduce falls in older women.

Identifying modifiable risk factors is essential for fall prevention, enabling the development of targeted interventions that can reduce fall risk and promote independence among older adults [5, 6]. Unlike non-modifiable factors, such as age or medical history, modifiable factors, like physical inactivity, poor balance, or low balance confidence, can be addressed through behavioral and exercise-based interventions [7]. Among these factors, balance confidence, an individual’s confidence in their ability to stay stable and balanced while performing everyday activities, such as getting dressed or climbing stairs, is a significant predictor of fall risk [3], independent of physical function (PF) – or the ability to perform activities of daily living [8, 9]. Balance confidence is particularly valuable as an outcome measure because it can be efficiently assessed using self-reported scales, enabling widespread adoption and implementation in clinical and community settings [10]. The Activities-specific Balance Confidence (ABC) scale is one of the most widely used patient-reported outcome measures to assess balance confidence with excellent reliability and validity for community-dwelling older adults across a wide spectrum of PF levels [10–13]. Clinically, it is used to assess baseline fall risk and changes in fall risk following an intervention [13–15].

Adults aged ≥ 85 years, known as the “oldest old,” are a rapidly growing, yet very understudied, proportion of the older adults included in fall prevention research [16]. Physical activity (PA) engagement, sedentary behavior, and their relationships to fall risk remain underexplored in women aged ≥ 85 years. Data collection from women assigned to the PA intervention arm of the Women’s Health Initiative Strong and Healthy (*WHISH*) pragmatic trial [17, 18] provided the opportunity to examine relationships of balance confidence with specific types of PA, e.g. strength training, walking, and sitting, and physical function (PF) in a large cohort of women aged 75 and over, half of whom were 85 and over.

Previous research has demonstrated that low balance confidence is associated with increased fall risk and lower levels of PA and PF in older adults [10, 19]. Individuals with low balance confidence may avoid PA due to fear of falling, potentially leading to further physical decline and elevated fall risk [20]. Conversely, limited engagement in PA may contribute to diminished balance confidence [21]. However, it remains unclear which specific types of PA (e.g. walking, strength training) are most strongly associated with balance confidence and fall risk. Additionally, there is limited understanding of how balance confidence relates to sedentary behavior, such as sitting time. While balance confidence and self-reported PF are strongly correlated among community-dwelling older women [22, 23], it is unclear how this relationship varies across different PF groups. Understanding these associations can help tailor interventions that target both the psychological and physical components of fall risk.

In this cross-sectional analysis, we leverage data collected in 2022 from Women’s Health Initiative Strong and Healthy (*WHISH*) intervention participants who had been receiving mostly print materials promoting PA recommendations from the Department of Health and Human Services (DHHS) [17, 18]. Our objective is to identify the association of strength training, walking, sitting time, and PF with low balance confidence and increased FR-ABC, specifically comparing these associations between women aged 75–84 years and those aged ≥ 85 years. By examining these relationships in a large, community-dwelling population of older women, this study aims to inform the selection of target populations for future interventions. It also lays the groundwork for subsequent longitudinal and interventional research in fall prevention.

## METHODS

### Study Population

In 2015, the Women’s Health Initiative Strong and Healthy (*WHISH*) trial randomly assigned over 24,000 women to a centrally delivered intervention providing national (DHHS) PA recommendations for older adults, i.e. aged 65+ [17, 18] through multiple remote channels, including quarterly newsletters and accompanying print materials, monthly automated telephone calls, monthly electronic mail (to participants who provided email addresses), and a website [17, 18]. Embedded within the Women’s Health Initiative (WHI) Extension Study—a continuation of the original WHI Clinical Trials and Observational Studies conducted between 1993–1998—the *WHISH* cohort includes geographically diverse older women from 40 clinical centers across the United States [24].

All *WHISH* participants were originally enrolled in the Women’s Health Initiative (WHI) Clinical Trials or Observational Study at 40 U.S. clinical centers between 1993 and 1998 at ages 50–79 years,[24] and subsequently consented to ongoing follow-up in the WHI-Extension Study in 2005 and again 2010. Of the 49,331 WHI participants who were eligible for the *WHISH* trial, 23,653 (of 24,657 assigned to the PA-Intervention) passively consented to receiving the PA intervention from the Stanford PA-Intervention site [25]. The current analysis is limited to women deemed to still be “active”, i.e. alive and receiving newsletters, in 2022 (N = 14,819) and who returned the annual survey that year. We analyzed data for the whole sample and stratified results by two age groups: 75–84 years old and 85 years and older. This age stratification aligns with existing literature distinguishing the “old-old” from “oldest-old” populations, groups that demonstrate distinct patterns in fall risk and functional decline [26].

### Fall Risk Classification

Fall risk classification (FR-ABC) was defined as an ABC score < 67%, a validated threshold previously shown to predict falls in older adults with 84% accuracy [22, 27]. The 2022 PA-Intervention survey included the ABC scale, a 16-item inventory that asks individuals to rate their balance confidence for performing activities with the question stem for each activity being “How confident are you that you will not lose your balance or become unsteady when you…” The individual rates the items on a 0–100% scale with 0 representing no confidence and a score of 100 relating to complete confidence. An overall score can then be calculated as a percent of overall balance confidence by adding all scores and dividing by the 16 total items. A score less than 67% classifies an individual as at risk for falls [22].

### Self-reported Strength Training, Walking, Sitting Time, and PF

Self-reported strength training, walking, sitting time, and PF were collected annually by mailed questionnaires.[28] For this analysis, we utilized the most recent data collected in 2022. PA was determined by a modified CHAMPS II PA Questionnaire for Older Adults[29, 30] which assesses weekly frequency, duration, and intensity (light to vigorous) of PA and asks participants to recall average effort per week in the past month. Activities surveyed include running, walking, water aerobics, tai-chi, yoga, and strength training sessions. Strength training was defined by doing moderate to heavy training, such as hand-held weights of more than 5 lbs, weight machines, or push-ups. Individuals were also asked to assess intensity of the activities performed. These tasks were then grouped by PA types which include strength training activities, aerobics (non-walking), and walking-based aerobics. A measure of all activity was also provided. Questions related to time spent sitting per day are also asked in the same form (WHI Form 521) as the CHAMPS II questionnaire. For each PA type, 3–4 groups were created representing low, moderate, and high levels.

PF was based on ten items from the RAND 36-item health survey, a well-validated measure of self-reported PF[31], which measures the ability to engage in moderate to vigorous activities (2 items), strength for tasks like lifting, carrying, stooping, bending, and stair climbing (4 items), the capacity to walk different distances without difficulty (3 items), and self-care (1 item). Scores range from 0–100 with a score of 0 denoting completely limited and 100 meaning no limitations in any activities and the scores were grouped into four categories: low (< 40; n = 970), moderate (40–59; n = 876), high (60–89; n = 2064), and very high (> 90; n = 1441) for analyses.

### Statistical Analyses

Descriptive analyses summarized baseline characteristics as of June 2023 and bivariate analyses examined associations between FR-ABC and strength training, walking, sitting time, and PF. For each characteristic, we estimated the mean and standard deviation of the ABC score. We used analysis of variance (ANOVA) tests to assess the overall significance of the difference in the mean of ABC scores and post-hoc tests on the mean differences and 95% confidence intervals (CI) in balance confidence scores for strength training, walking, sitting time, and PF group relative to the reference category (e.g., < 1.5 active hours, < 40 PF score). To correlate FR-ABC with strength training, walking sitting time, and PF level, we calculated the proportion of women at risk within category. We performed chi-squared tests to test the overall group difference in proportions. We used a Poisson model with robust error variance[32] to estimate the relative risk (RR) with 95% confidence intervals of FR-ABC by strength training, walking, sitting time, and PF categories, setting the reference groups to 0 hours/week for each PA type, < 5 hours/day of sitting time, and < 40 for PF category. Stratified analyses were also performed by age group (75–84 years and 85 years and older). Statistical significance was set at alpha = 0.05. No formal power calculation was performed for this cross-sectional analysis as this was a secondary analysis of existing trial data. We used SAS version 9.4 (SAS Institute Inc, Cary NC) to conduct all statistical analyses.

## Results

### Baseline Characteristics

Table 1 presents the demographic and functional characteristics of the 8,916 *WHISH* participants included in this analysis, overall and stratified by FR-ABC status and age group. The mean age of participants was approximately 84.9 years, with 50.4% aged 75–84 years and 49.6% aged ≥ 85 years. Based on an ABC score < 67%, 35.1% of participants (N = 3130) were classified as at risk for falls, i.e. prevalent FR-ABC, including 22.7% of women aged 75–84 (N = 1036) and 47.4% (N = 2094) of women aged 85 and older. Most participants (64.7%) reported no strength training, including 62.5% of women aged 75–84 and 67.0% of those aged 85 and over (Table 2). While 77.4% reported some walking activity, 22.6% reported none, and only 29.0% reported ≥ 4.25 hours per week. Sitting ≥ 8 hours per day was reported by 38.0% of participants, including 36.2% of women aged 75–84 and 39.9% of those aged ≥ 85.

### Fall Risk Classification, Strength Training, Walking, and Sitting Time

**Figure 1:**
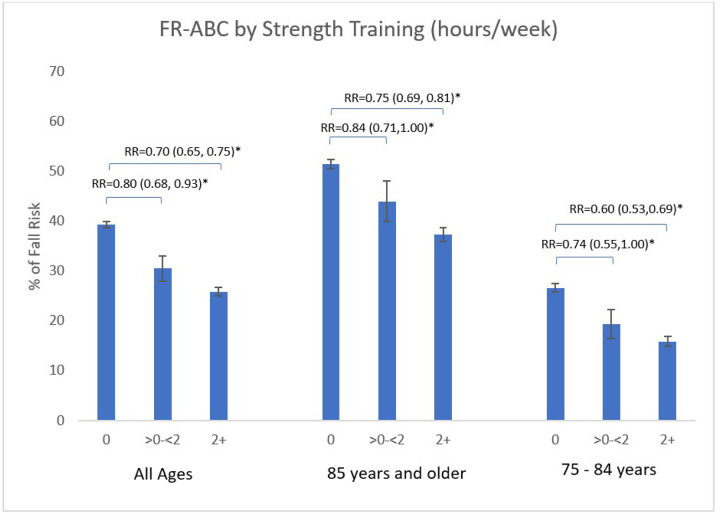
Fall risk classification (FR-ABC) by strength training hours per week stratified by age group

**Figure 2:**
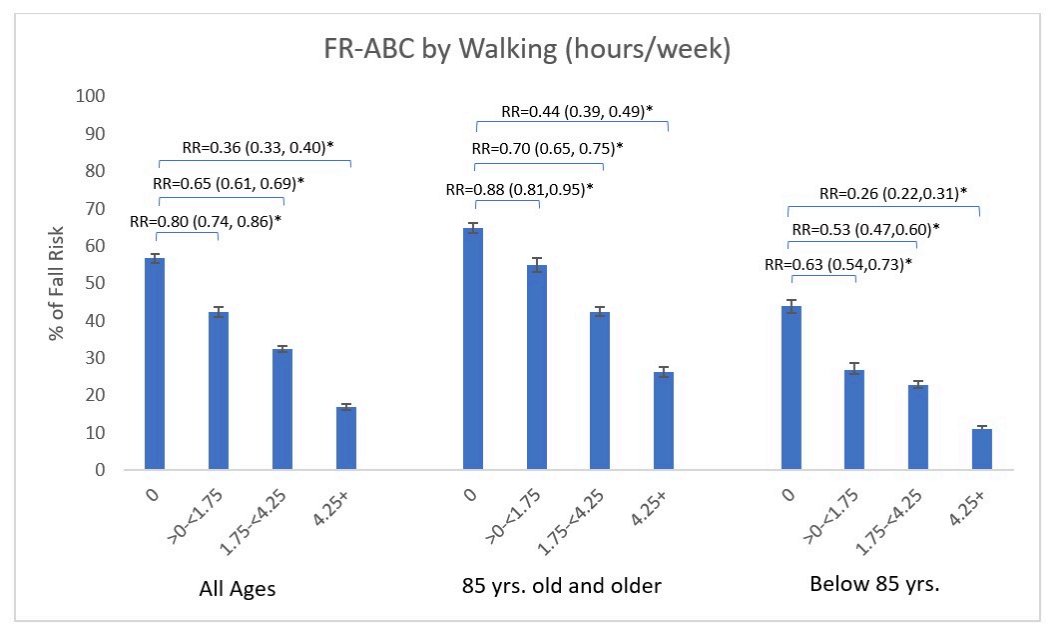
Fall risk classification (FR-ABC) by walking hours per week stratified by age group

Fall risk was significantly lower among participants who engaged in more strength training (p < 0.0001; Fig. 1) and walking (p < 0.0001; Fig. 2), demonstrating a dose-dependent relationship. Among women who reported no strength training, FR-ABC was 39.3%. In comparison, those performing ≥ 2 hours per week had a FR-ABC of 25.8%, corresponding to a 30% FR-ABC risk reduction (p < 0.0001; RR = 0.70, 95% CI: 0.65–0.75; Fig. 1). Among women aged 75–84 years, those reporting no strength training had a FR-ABC of 26.6%, compared to 15.9% among those performing ≥ 2 hours per week, corresponding to a 40% FR-ABC risk reduction (RR = 0.60, 95% CI: 0.53–0.69; p < 0.0001). Among women aged ≥ 85 years, those reporting no strength training had a FR-ABC of 51.4%, compared to 37.4% among those performing ≥ 2 hours per week, corresponding to a 25% FR-ABC risk reduction (RR = 0.75, 95% CI: 0.62–0.74; p < 0.0001).

Those reporting 0 hours of walking per week had a FR-ABC of 56.7% compared to 17.0% among those reporting ≥ 4.25 hours of walking per week, corresponding to a 64% FR-ABC risk reduction (p < 0.0001; RR = 0.36, 95% CI: 0.33–0.40). Among women aged 75–84 years, FR-ABC was 43.9% in those reporting no walking and 11.2% in those walking ≥ 4.25 hours/week, corresponding to a 74% FR-ABC risk reduction (RR = 0.26, 95% CI: 0.22–0.31; p < 0.0001). In those ≥ 85 years, FR-ABC was higher across the same walking categories (64.8% vs 26.4%), corresponding to a 56% FR-ABC risk reduction (RR = 0.44, 95% CI: 0.39–0.49; p < 0.0001; Fig. 2).

**Figure 3:**
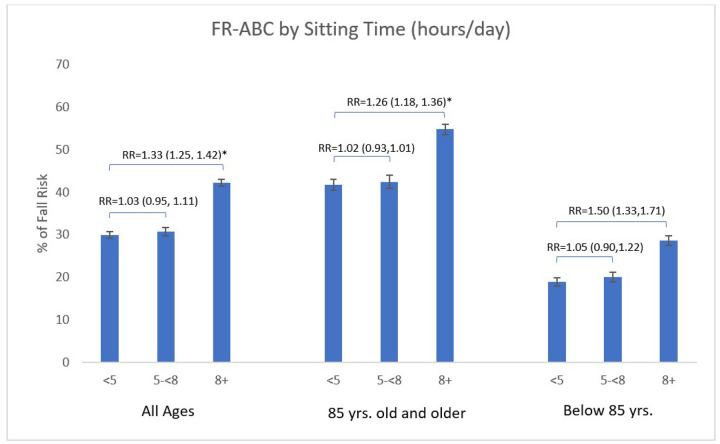
Fall risk classification (FR-ABC) by sitting hours per day stratified by age group

FR-ABC was also significantly associated with sitting time (p < 0.0001; Fig. 3). Women who reported sitting ≥ 8 hours per day had FR-ABC of 42.2%, compared to 29.9% among those sitting < 5 hours per day, corresponding to a 41% FR-ABC risk reduction (RR = 1.41, 95% CI: 1.32–1.51; p < 0.0001). There was no significant difference in FR-ABC between those sitting 5–8 hours per day and those sitting < 5 hours (RR = 1.03, 95% CI: 0.95–1.11; p = 0.51).

### Fall Risk Classification and Physical Function

**Figure 4:**
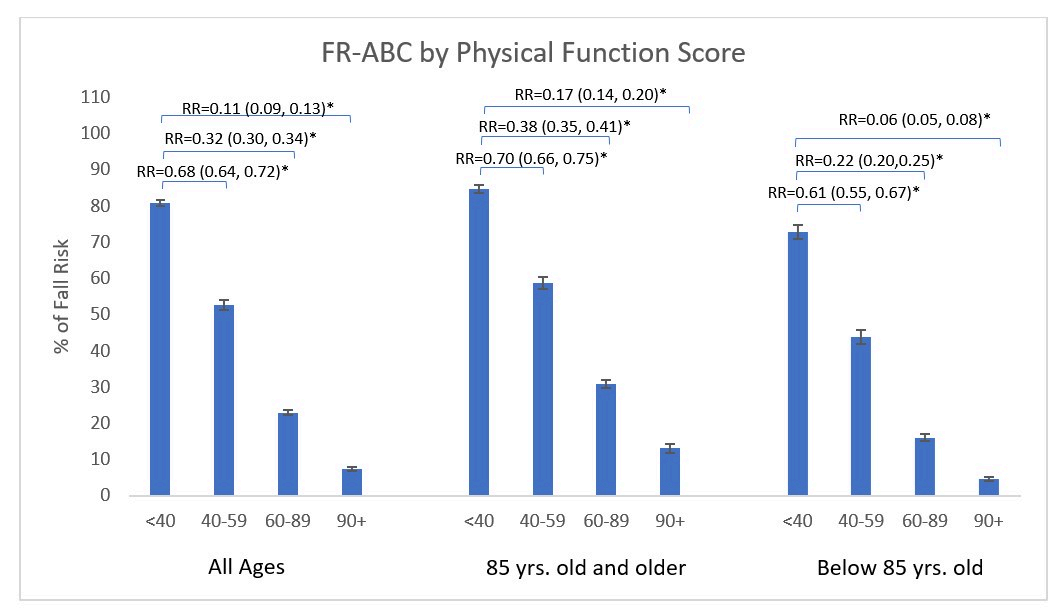
Fall risk classification (FR-ABC) by physical function score stratified by age group

Higher PF scores were significantly associated with lower FR-ABC (p < 0.0001; Fig. 4). Participants with low PF (PF score < 40) had FR-ABC of 80.8%, compared to 7.4% among those with very high PF (PF ≥ 90), corresponding to an 89% risk reduction (RR = 0.11, 95% CI: 0.09–0.13; p < 0.0001; Fig. 3). Among women aged 75–84 years, FR-ABC was 76.2% in those with low PF and 14.2% among those with very high, corresponding to an 81% FR-ABC risk reduction (RR = 0.19, 95% CI: 0.16–0.23; p < 0.0001; Fig. 3). In women aged ≥ 85 years, FR-ABC was 88.6% and 23.7% across the same PF categories, corresponding to a 73% FR-ABC risk reduction (RR = 0.27, 95% CI: 0.23–0.31; p < 0.0001; Fig. 3).

## Discussion

In this large, cross-sectional study of nearly 9,000 women aged 75 and older, we observed a clear dose-dependent relationship between FR-ABC and both strength training and walking, with the relationship more pronounced among woman aged 75–84. Our study highlights the importance of distinguishing the “oldest old”, the fastest growing segment of the population, for whom data on PA and fall risk remain limited.

Our findings may underscore the value of strength training and walking specifically, among the oldest old, to reduce FR-ABC. This is especially important given that the US Physical Activity Guidelines recommend strength training two times per week [18] and only 35% of our sample reported strength training more than 0 hours per week, with comparable participation in those 75–84 years old and those 85 years and over (37% vs 33%). This is higher than the national average for women over 75–84 (16.3%) and women 80 and over (9.6%) reporting participating in strength training [33], though this may be because most estimates ask if participants are meeting the recommended guidelines of at least 2 times per week versus total time. In addition, our sample had been receiving PA intervention materials for 6 years prior to this survey which may have led to increased reporting and/or participation in strength training [25]. Though not observed in this study, national estimates of strength training are nearly half between women aged 75–84 and those aged ≥ 85 years, highlighting the oldest old as a potential priority group for targeted resistance training messaging and interventions. Importantly, strength training does not require access to gyms or heavy equipment. It can be safely and effectively performed at home using body weight or resistance bands, making it an accessible strategy for older adults with mobility or transportation limitations, and is a key component of the *WHISH* intervention.

We also found that increased sitting time was associated with greater FR-ABC, though the increase in relative risk was only significant for those sitting 8 hours or more per day compared to those sitting less than 5 hours per day. There was no significant difference in FR-ABC between women sitting 5–8 hours per day and those sitting less than 5, suggesting a potential threshold effect, where FR-ABC becomes elevated only at higher levels of sedentary behavior. When stratified by age, this association was more pronounced among women aged 75–84 years, consistent with the patterns observed for strength training and walking. These findings reinforce the idea that sedentary behavior may contribute to FR-ABC in older adults and support public health recommendations to reduce sitting time [34–36]. Importantly, the stronger associations seen in the younger age group could reflect greater variability in behavior or functional reserve, whereas older adults with higher FR-ABC may already be limited in their ability to modify sitting time. Together, these results suggest that reducing sedentary behavior, alongside increasing PA, may represent an actionable strategy for fall prevention, particularly among women in their mid-to-late 70s.

Our findings demonstrate a strong, graded cross-sectional association between PF and FR-ABC, with higher PF scores consistently linked to lower likelihood of FR-ABC. Women with very high PF (≥ 90) had an 89% lower likelihood of FR-ABC compared to those with low PF (< 40), a pattern that held across both age groups. These results align with prior studies showing that greater functional capacity is protective against falls in older adults and further highlight PF as a critical target for fall prevention strategies[37–40]. While FR-ABC decreased across PF categories in both age groups, the association was more pronounced among women aged 75–84 years. This attenuation in the oldest group may reflect a ceiling effect in risk among women with already reduced capacity, or other unmeasured factors that contribute to falls in very late life (e.g., cognitive impairment, frailty, or medical complexity). These results reinforce the value of preserving and enhancing PF to mitigate FR in later life. Given that PF is modifiable through structured exercise and rehabilitation, especially strength and balance training, it remains a promising and practical target for interventions. Our findings also suggest that identifying and supporting women with low PF, particularly those in their late 70s and early 80s, may be an effective strategy for reducing the population burden of falls.

This study has several limitations. First, the design is cross-sectional and therefore, does not allow for determining cause and effect between FR-ABC and strength training, walking, sitting time, and PF outcomes nor does it allow for an understanding of how FR-ABC changes over time. It is unclear whether higher PA and PF leads to lower FR-ABC or if those with lower FR-ABC tend to engage in more PA, sit less, and maintain higher PF in our population. Second, our outcome measures are based on self-report. While the ABC scale is widely validated, it is still a subjective measure and may not fully capture functional balance capacity. Participants were also asked to mail survey responses back which may result in a response bias, though we achieved ~ 60% response rate. Lastly, this analysis does not include participants’ prior fall history, which is likely to be strongly associated with FR-ABC and may also influence self-reported PA and PF. Future studies should explore measuring FR-ABC longitudinally to understand how it changes for individuals over time. Additionally, reliance on self-reported data may introduce bias.

Despite these limitations, this study has three strengths not found in previous studies. First, our dataset includes a large sample of participants spread geographically across the US which enhances the generalizability of the results to older women. Second, this study provides valuable insight into FR-ABC within the oldest old, an age group often underrepresented yet most vulnerable to falls. Lastly, we examine resistance training and walking, providing greater insight into the relationship between specific types of PA and FR-ABC.

These findings may inform PA messaging campaigns and fall prevention strategies aimed at engaging older women, especially those in their late 70s who appear most responsive to behavior change. Given that women aged ≥ 85 years showed lower participation in strength training and smaller relative risk reductions, this group may require tailored approaches that account for mobility limitations and comorbidities. Additionally, the fact that most women in this cohort reported no strength training underscores the need for greater public awareness and access to age-appropriate resistance training opportunities.

## Conclusion

In this large, cross-sectional analysis of nearly 9,000 women aged 75 and older, greater participation in strength training and walking, lower sitting time, and higher PF were each significantly associated with lower FR-ABC. These associations followed clear dose-dependent patterns and were generally more pronounced among women aged 75–84 years compared to those aged ≥ 85 years. Notably, the strongest differences in lower FR-ABC were observed with higher levels of PF. These findings highlight the interconnected roles of PA, sedentary behavior, and functional status in FR-ABC and underscore the potential of targeting these modifiable factors in future interventions. Given the limited participation in strength training, particularly among the oldest old, there is a clear opportunity for public health messaging and programming to focus on engaging this population. Future research should explore how changes in these behaviors influence fall risk over time, especially in high-risk groups.

## Figures and Tables

**Table 1:** Fall risk classification (FR-ABC) of *WHISH* Respondents by individual characteristics (N = 8,916^a^)

	Total(N = 8916)		Classified as Fall Risk (FR-ABC) (N = 3130)		Age 75–84 years (N = 4493)		Classified as Fall Risk (FR-ABC) (N = 1036)		Age ≥ 85 years (N = 4422)		Classified as Fall Risk (FR-ABC) (N = 2094)	
Category	N	%	N	%	N	%	N	%	N	%	N	%
Race												
White	7698	86.3	2718	35.3	3777	84.1	858	22.7	3920	88.7	1860	47.5
Black/African American	795	8.9	274	34.9	464	10.3	120	25.9	331	7.5	159	48.0
Asian	194	2.2	52	27.1	112	2.5	18	16.1	82	1.9	34	41.5
American Indian/Alaska Native	22	0.3	7	36.8	17	0.4	5	29.4	5	0.11	3	60
Native Hawaiian/Other Pacific Islander	3	0.03	2	66.7	2	0.04	2	100	1	0.02	0	0
More than one race	106	1.2	36	37.1	64	1.4	19	29.7	42	0.95	17	40.5
Unknown/Not reported	98	1.1	11	45.8	57	1.3	14	24.6	41	0.93	21	51.2
Ethnicity												
Non-Hispanic/Latino	8538	95.8	3008	35.2	4274	95.1	982	23.0	4264	96.43	2026	47.51
Hispanic/Latino	360	4.1	120	33.3	206	4.6	54	26.2	153	3.46	66	43.14
Unknown/Not reported	18	0.2	2	11.1	29	0.7	0	0	5	0.11	2	40
Education												
College degree or higher	4695	52.7	1407	30.0	2501	55.7	458	18.3	2194	49.6	949	43.3
School after high school	2915	32.7	1100	37.3	1457	32.4	389	26.7	1458	33.0	711	48.8
< High school/GED	1253	14.1	606	48.4	506	11.3	182	36.0	746	16.9	424	56.8
Missing	53	0.6	17	32.1	29	0.65	7	24.1	24	0.5	10	41.7
Use of Walking Aids												
No aids	8244	92.5	2622	31.8	4300	95.7	909	21.1	3943	89.2	1713	43.4
Cane	387	4.3	261	67.4	135	3	82	60.7	252	5.7	179	71.0
Walker	257	2.9	224	87.2	50	1.1	38	76	207	4.7	186	89.9
Wheelchair	13	0.15	12	91.7	5	0.1	4	80	8	0.18	8	100
Missing	15	0.17	11	73.3	3	0.07	3	100	12	0.27	8	66.7

a Out of 9124 participants, 8916 participants have answered at least 12 ABC items

**Table 2:** Fall risk classification (FR-ABC) of *WHISH* Respondents by self-reported weekly participation in strength training and walking and daily sitting hours

	Total (N = 8916)		Classified as Fall Risk (FR-ABC) (N = 3130)		Age 75–84 years (N = 4493)		Classified as Fall Risk (FR-ABC) (N = 1036)		Age ≥ 85 years (N = 4422)		Classified as Fall Risk (FR-ABC) (N = 2094)	
Category	N	%	N	%	N	%	N	%	N	%	N	%
Strength hours/week												
0	5772	64.7	2270	39.4	2810	62.5	748	26.6	2961	67.0	1522	51.4
> 0-<2	331	3.7	101	30.5	181	4.0	35	19.3	150	3.39	66	44
2+	2688	30.2	694	25.8	1443	32.1	229	15.9	1245	28.2	465	37.4
Missing	125	1.4	65	52	59	1.3	24	40.7	66	1.5	41	62.1
Walking hours/week												
0	2018	22.6	1144	56.7	782	17.4	343	43.9	1236	28.0	801	64.8
> 0–<1.75	1241	13.9	525	42.3	559	12.4	150	26.8	682	15.4	375	55.0
1.75–<4.25	2949	33.1	958	32.5	1497	33.3	341	22.8	1452	32.8	617	42.5
4.25+	2583	29.0	438	17.0	1596	35.5	178	11.2	986	22.3	260	26.4
Missing	125	1.4	65	52	59	1.3	24	40.7	66	1.5	41	62.1
Sitting hours/day												
< 5	3110	34.9	931	29.9	1615	35.9	307	19.0	1495	33.8	624	41.7
5–<8	2324	26.1	715	30.8	1209	26.9	242	20.0	1114	25.2	473	42.5
8+	3388	38	1431	42.2	1626	36.2	466	28.7	1762	39.9	965	54.8
Missing	94	1.05	53	56.4	43	0.96	21	48.8	51	1.2	32	62.8

## Data Availability

The datasets used and/or analyzed during the current study are available from the corresponding author on reasonable request. Data sharing is subject to approval by the Women’s Health Initiative and adherence to the WHI data sharing policies.
